# Modality-specific attention in foraging bumblebees

**DOI:** 10.1098/rsos.150324

**Published:** 2015-10-07

**Authors:** Vivek Nityananda, Lars Chittka

**Affiliations:** 1 Department of Biological and Experimental Psychology, School of Biological and Chemical Sciences, Queen Mary University of London, Mile End Road, London E1 4NS, UK; 2 Institute of Neuroscience, Newcastle University, Henry Wellcome Building for Neuroecology, Framlington Place, Newcastle Upon Tyne NE2 4HH, UK

**Keywords:** *Bombus terrestris*, crossmodal attention, divided attention, foraging, predation

## Abstract

Attentional demands can prevent humans and other animals from performing multiple tasks simultaneously. Some studies, however, show that tasks presented in different sensory modalities (e.g. visual and auditory) can be processed simultaneously. This suggests that, at least in these cases, attention might be modality-specific and divided differently between tasks when present in the same modality compared with different modalities. We investigated this possibility in bumblebees (*Bombus terrestris*) using a biologically relevant experimental set-up where they had to simultaneously choose more rewarding flowers and avoid simulated predatory attacks by robotic ‘spiders’. We found that when the tasks had to be performed using visual cues alone, bees failed to perform both tasks simultaneously. However, when highly rewarding flowers were indicated by olfactory cues and predators were indicated by visual cues, bees managed to perform both tasks successfully. Our results thus provide evidence for modality-specific attention in foraging bees and establish a novel framework for future studies of crossmodal attention in ecologically realistic settings.

## 1. Introduction

Attention is a limited resource: the restriction of cognitive processing to a subset of all available stimuli is a defining feature of attention [1,2]. The resulting limitation in processing means that when two tasks need to be performed simultaneously, performance on both tasks may suffer compared with when they are carried out separately [3,4]. Such a decrement in performance has been observed for humans in several experiments (e.g. [5,6]) and is often observed during visual search for multiple targets [7,8]. Non-human animals have to perform multiple tasks or search for multiple targets simultaneously and they often appear to have a similar reduction in performance [9,10]. Jays (*Cyanocitta cristata*), for example, have a reduced probability of detecting a peripheral object (such as a predator) when attending to a difficult central task [11], and also have lower success rates when searching for two different prey types simultaneously [12]. Recent studies have investigated this possibility in bees [13,14]. While foraging, bees have to choose between highly and poorly rewarding flowers while simultaneously avoiding cryptic predators like crab spiders [15]. The cognitive demands of these simultaneous tasks have been investigated experimentally by presenting bees with both tasks in a set-up with robotic ‘spiders’ that could simulate a predatory attack [13,15]. In such a set-up, bees manage to perform both tasks simultaneously if the spiders are different in colour to the background but not if they are similar in colour. They seem do this by using the colour contrast of the spiders to detect the shapes of the spiders but subsequently recognize the shapes independent of colour [16]. This suggests that bees might have a search image for the spiders. It has also similarly been suggested that bees have search images for flowers [17]. Their success on this task might thus involve comparisons of multiple search images. These results indicate that bees, while capable of divided attention in some cases, cannot always simultaneously perform two difficult tasks, or not with equal efficiency [13,15].

Multiple tasks presented in different modalities can sometimes be performed simultaneously [18–23]. These results indicate that attentional limitations might not extend across modalities. It has instead been suggested that for these tasks, attention could be modality-specific, i.e. there are separate cognitive resources for processing different modalities [18,23,24]. This does not, however, apply across all tasks [5,25–30]. For certain tasks, targets presented in one modality prevent immediate recognition of targets in another modality and allow recognition only after a delay [3,29]. There also appears to be some exogenous attention capture when a stimulus is presented in one modality that contributes to crossmodal attentional limitation [30], and research into the use of cell phones while driving also indicates a cost to dividing attention between vision and audition [26,31]. Thus, attention can also be restricted by the presence of multiple crossmodal tasks and crossmodal interference or facilitation could be task-specific. It is, therefore, important to test the effect of crossmodal presentation of tasks on divided attention on a case-by-case basis.

Crossmodal effects on cognitive processing have rarely been studied in non-human animals [32–36], despite the fact that similar crossmodal processing is likely to play a vital role for some non-human species. Bumblebees, for example, must survive in an environment full of sensory stimuli in different modalities. They forage from flowers that present them multiple cues in multiple modalities—both visual and olfactory—and can use cues in either modality to discriminate highly rewarding flowers from less rewarding ones [37,38]. When both cues are present, bees are better at discriminating rewarding targets [32] and olfactory cues have been shown to reduce uncertainty about visual discriminations [33] and reduce loss of accuracy for visual signals under low light conditions [34]. This suggests that the presence of multimodal cues enhances their performance on a single task. Similar results have been found in spiders, where vibratory stimuli facilitate colour discrimination [36] and cause female wolf spiders to be more responsive to male visual signals [35], though they also become less likely to notice predatory visual signals [35].

No study, however, has so far investigated whether attending to different tasks in the same modality or in different modalities results in different demands on cognitive processing for foraging bumblebees. In our study, we investigated whether bumblebees (*Bombus terrestris*) differ in their abilities for divided attention when tasks were presented in either the same or differing modalities. The bees had to simultaneously perform two ecologically relevant tasks: a foraging task where they had to distinguish highly rewarding from lower rewarding flowers and a predator avoidance task where they had to distinguish flowers with spiders in favour of flowers without spiders. In different experiments, the highly rewarding and lower rewarding flowers differed in either colour or odour cues while ‘dangerous’ flowers were always indicated with the visual cue of the spiders.

**Figure 1.**
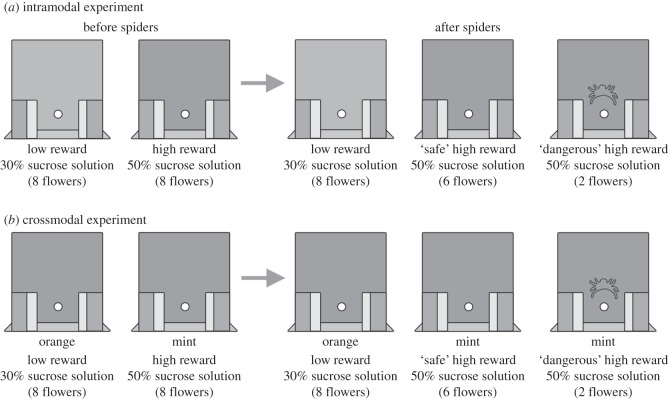
Experimental paradigms. Illustration of the artificial flowers with the feeding holes and the clamps in front of them that could simulate a predatory attack. (*a*) In the intramodal experiment, bees were first trained to distinguish artificial flowers of two similar shades of yellow (grey in our figure). After they were trained on this task, cryptic models of crab spiders were introduced on two of the highly rewarding flowers. (*b*) In the crossmodal experiment, bees were first trained to distinguish artificial flowers of the same shade of yellow (grey in our figure) that had either mint (for the highly rewarding flowers) or orange (for the low rewarding flowers) odour cues provided from behind the flower. The odour cues are indicated in the figure under each artificial flower. After they were trained on this task, cryptic models of crab spiders (silhouettes in the figure) were introduced on two of the highly rewarding flowers. The odour cues were still provided and associated with the same level of reward.

We were looking to test the hypothesis that bees have attention-like processes specific to each modality, i.e. that each modality is processed separately. This would predict that, if the two tasks were both visual (intramodal condition; figure 1*a*), they would receive divided amounts of attention from the visual system, whereas if one task was visual and the other olfactory (crossmodal condition; figure 1*b*), each would receive the undivided attentional resources available to each modality. Performance in the latter condition would then be predicted to be better than performance in the condition with two visual tasks. The null hypothesis is that bees have common attentional resources that are divided between the visual and olfactory modalities. Performance when faced with two tasks would then be predicted to be similar regardless of the modality of the two tasks.

The difficulty of both tasks in these experiments is an important consideration for the above hypotheses. If the second task was easier in one modality than the other, it would be expected that the bees could perform two tasks rather than one in the crossmodal condition simply because the tasks were easier in this condition compared with the other. In addition, if one stimulus was more salient than the other in any of the tasks, we could see overshadowing, where the more salient stimulus prevents the learning of a less salient one. If this affected the crossmodal tasks, then we might, for example, see that a more salient visual target prevents the bee from learning the olfactory task. We, therefore, investigated the difficulty of the tasks in our experiments by measuring how long it took the bees to learn each task and the final accuracy levels they reached in each task.

## 2. Material and methods

### 2.1 Animals

Experiments were conducted on bumblebee (*B. terrestris*) workers from three different colonies. All colonies were obtained from Syngenta Bioline (Weert, The Netherlands). We tagged the bees individually with coloured Opalith number tags (Christian Graze KG, Weinstadt-Endersbach, Germany) and transferred them in darkness to one chamber of a bi-partite wooden box. The second chamber was filled with cat litter to allow the bees somewhere to discard waste. Bees were fed every other day with 50% sucrose solution and pollen except on experimental days to maintain motivation.

### 2.2 Experimental set-up

Bee colonies were connected to the experimental arena by a Perspex^r^ tube. The tubes had slits in their sides that enabled us to insert cardboard or Perspex^r^ barriers. These barriers were used to control the access into the arena and allow individual bees to enter the arena. The flight arena consisted of a wooden box (dimensions: 1×0.72 m and 0.73 m high) covered with a UV-transparent Plexiglas lid. Illumination was provided by high frequency fluorescent lighting (TMS 24F lamps with HF-B 236 TLD ballasts, Philips, The Netherlands, fitted with Activa daylight fluorescent tubes, Osram, Germany) that mimicked natural daylight including a UV component and flickered at around 4.2 KHz, above the frequency detectable by bees [39,40]. The wall of the arena farthest from the entrance contained sixteen feeding balconies (each 40×60 mm) arranged in a four by four grid against a grey background. These balconies could be fitted with artificial flowers consisting of a coloured rectangular acrylic card (7 mm square and 1 mm thick). All the artificial flowers consisted of a feeding hole 10 mm above the feeding balcony through which the bees could access foraging rewards present behind the arena wall. Foraging rewards were delivered through syringes connected to a pump system as described previously [13,15]. Each balcony was also fitted with a robotic ‘crab spider’, mimicking the natural scenario of flower meadows where crab spiders wait on flowers ready to attack pollinators [15]. The spider mechanism enables us to harmlessly and briefly capture the bee with sponge-covered pincers, simulating a predation attempt. A visual image was present on all flowers on which we simulated a predation attempt (see the following section).

### 2.3 Intramodal experiment

For the intramodal experiment, seven bees were pre-trained for at least one day to forage for drops of sucrose with feeding balconies having no artificial flowers behind them. Once they began foraging from the artificial flowers over multiple foraging flights, individual bees were trained in two consecutive training phases: before and after the introduction of spiders (figure 1*a*).

In the ‘before-spider phase’, bees were trained to discriminate between artificial flowers of two different shades of yellow that were chosen to be very similar to each other as perceived by the bees (see [13] for details of reflectance measurements). The darker yellow artificial flowers had rewards of 50% sucrose solution (volume/volume), whereas the lighter yellow flowers had rewards of 30% sucrose (volume/volume). These represent relative concentrations that bumblebees can discriminate [41]. The choices of the bees were noted and bees were allowed to return to the colony after a foraging flight. Between successive trials (foraging bouts), the positions of the high- and low-reward flowers were changed according to a previously decided random order and the corresponding syringes, that delivered the solution to the flowers, were also changed. To prevent odour cues affecting behaviour on subsequent trials, the feeding balconies were cleaned with ethanol and water and wiped dry. Bees were trained for a minimum of 100 choices and training continued until they successfully chose the highly rewarding flowers significantly above chance (greater than 14 out of 20 choices; binomial test, *p*=0.04). If the bees failed to meet this criterion after 150 choices, the trials were terminated.

Subsequent to training, dark yellow models of crab spiders (length=12 mm, made from Gedeo Crystal resin; see [15] for details of the colour reflectance of the spiders and the flowers) were fixed above the feeding holes of two (of eight) of the high-rewarding artificial flowers that were randomly chosen according to a previously set order. Bees were then allowed to approach the artificial flowers and their choices were noted. We simulated a predation attempt every time a bee probed a flower with a spider image. These predation attacks did not injure any of the bees and they subsequently continued flying and foraging. Bees were allowed to return to the colony after a foraging flight. As described above, between successive flights, the feeding balconies were cleaned with ethanol and water and wiped dry to prevent odour cues interfering with the next trial. The positions of the highly rewarding and lower rewarding flowers as well as the positions of the spiders were also changed according to a previously decided random order, as were the corresponding reward delivery syringes. Bees were allowed to continue to forage until they had chosen the highly rewarding flowers significantly above chance (greater than 14 of the last 20 choices, binomial test, *p*=0.04) after a minimum of 100 choices. If they did not meet this criterion after a 100 choices, training continued until they did, up to a maximum of 150 choices.

### 2.4 Crossmodal experiment

The crossmodal experiment (figure 1*b*) was run (10 bees tested) similarly to the intramodal experiment except that the high- and low-reward flowers differed in odour rather than colour—all artificial flowers were of the darker yellow colour. Glass vials (volume = 9.5 ml, 4 cm tall, 1.5 cm diameter) containing 10 μl of mint essential oils (Essential Oils Direct, Oldham, UK) diluted in 1:100 pentane were placed behind the back wall of the arena just under the feeding holes of the high-rewarding flowers and glass vials containing 10 μl of orange essential oils (Essential Oils Direct) diluted in 1:100 pentane were placed under the feeding holes of the low rewarding artificial flowers. These vials served to provide odour cues to the bees but placing them behind the flowers ensured that the bees did not have actual contact with the chemicals and reduced the possibility that bees could bring odour cues back to the nest [42]. The arena was ventilated in between foraging flights by removing the Plexiglas lid and turning on a fan for 1 min to reduce any lingering odour cues in the arena.

### 2.5 Data analysis

To investigate whether the odour and colour tasks were comparable in difficulty we compared the proportion of high-rewarding choices in the colour and odour tasks as well as the number of choices taken to learn each task in the before-spider phase using Mann–Whitney tests. To investigate the performance of the bees on the training tasks, we compared the mean proportion of choices for highly rewarding flowers in the first 30 choices and the last 30 choices of the bees within each phase using Wilcoxon signed-rank tests for both experiments separately. Comparing these within the before-spider phases revealed whether the bees learned to use either the colour or odour cues to discriminate between the flowers, whereas comparing these in the after-spider phase revealed whether the bees still chose the same proportion of highly rewarding flowers after the introduction of spiders. We also compared the proportion of high-reward choices and spider choices to chance proportions (0.5 for the high-reward choices; 0.125 for the spiders) to see if the bees successfully chose the high-reward choices above chance and managed to avoid choosing the spiders above chance. To compare across experiments, we ran a Friedman’s two-way ANOVA comparing the number of choices of highly rewarding flowers across individuals with the point in the time course (first 30 choices and last 30 choices) and the modality condition (intramodal or crossmodal) as factors. We ran this ANOVA separately for the choices before the spiders were introduced and after the spiders were introduced.

## 3. Results

### 3.1 Difficulty of tasks

To assess if the initial tasks before the introduction of spiders differed in their difficulty in the intramodal and crossmodal experiments, we compared the number of choices taken to learn the task and the final accuracy levels reached on the tasks. We found that the number of choices taken to learn the task without spiders did not differ significantly for the visual and odour tasks (Mann–Whitney test, *U*(16)=24.5, *Z*=−1.549, *p*=0.315). The mean proportion of highly rewarding flowers chosen in the last 30 choices also did not differ significantly for the visual and the olfactory tasks (Mann–Whitney test, *U*(16)=17.5, *Z*=−1.746, *p*=0.088) and was around 0.67 for both tasks.

### 3.2 Intramodal experiment

**Figure 2.**
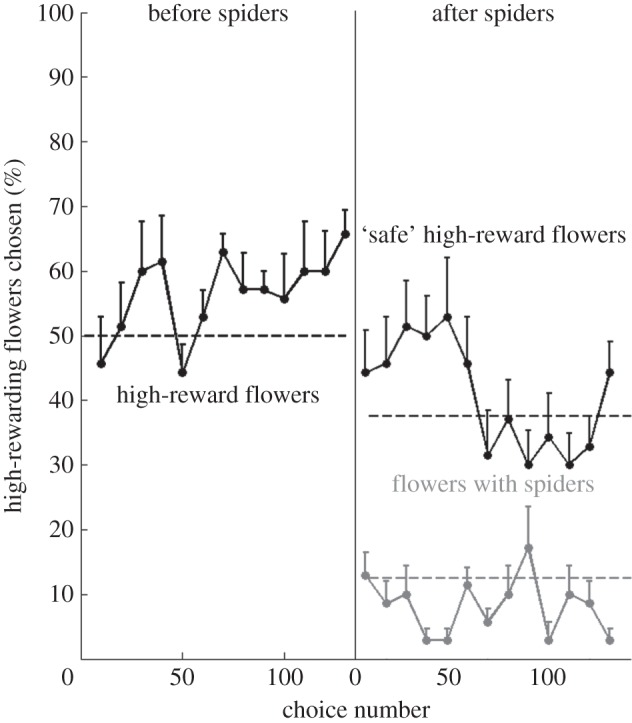
Intramodal experiment: bees fail to perform foraging and predator avoidance tasks simultaneously. The mean percentage of high-reward flowers with (grey line) and without (black lines) spiders chosen for consecutive non-overlapping blocks of 10 choices over the course of the experiment. Error bars indicate standard error values. The vertical line divides the data into choices before and after the spiders were introduced. Dashed lines represent chance levels of choices for high-reward flowers with (grey dotted line) and without (black dotted lines) spiders.

In their first 30 choices, bees failed to learn to differentiate the different yellow flowers and to approach highly rewarding flowers preferentially (figure 2, before spiders, one-sample Wilcoxon signed-rank test, *Z*=−0.736, *p*=0.462). By the last 30 choices before the introduction of the spiders, however, all bees learnt to use the colour cues to approach the highly rewarding flowers over the lower rewarding flowers significantly above chance (figure 2, before spiders; one-sample Wilcoxon signed-rank test, *Z*=−2.646, *p*=0.008). The mean number of highly rewarding flowers chosen was also significantly different in the last 30 choices compared with the first 30 choices (figure 2, before spiders; Wilcoxon signed-rank test, *Z*=−2.371, *p*=0.018). The bees took an average of 130 (±31.9 s.d.) choices distributed over 3 (±1.4 s.d.) foraging bouts to learn the task.

After the introduction of the spiders, the bees still chose the highly rewarding flowers significantly above chance in their first 30 choices (figure 2, after spiders; one-sample Wilcoxon signed-rank test, *Z*=−2.201, *p*=0.028). However, they failed to significantly avoid spiders in these first 30 choices (figure 2, after-spiders; one-sample Wilcoxon signed-rank test, *Z*=−1.020, *p*=0.308). In their last 30 choices after the introduction of the spiders, the bees chose flowers with spiders on them significantly below chance levels (figure 2, after spiders; one-sample Wilcoxon signed-rank test, *Z*=−2.205, *p*=0.027). They, however, failed to choose the highly rewarding flowers significantly above chance in these last 30 choices (figure 2, after spiders; one-sample Wilcoxon signed-rank test, *Z*=−0.679, *p*=0.497). They were thus unable to perform both visual tasks simultaneously.

### 3.3 Crossmodal experiment

**Figure 3.**
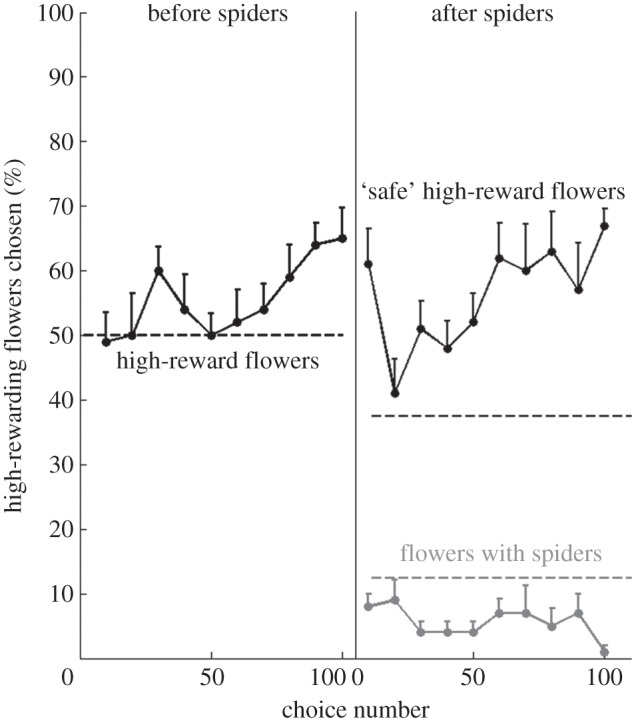
Crossmodal experiment: bees can perform foraging and predator avoidance tasks simultaneously. The mean percentage of high-reward flowers with (grey line) and without (black lines) spiders chosen for every consecutive non-overlapping block of 10 choices over the course of the experiment. Error bars indicate standard error values. The vertical line divides the data into choices before and after the spiders were introduced. Dashed lines represent chance levels of choices for high-reward flowers with (grey dotted line) and without (black dotted lines) spiders.

In the crossmodal experiment, bees did not differentiate between the highly rewarding flowers and the lower rewarding flowers in the first 30 choices they made (figure 3, before spiders; one-sample Wilcoxon signed-rank test, *Z*=−0.776, *p*=0.438). They did, however, choose highly rewarding flowers significantly above chance in the last 30 choices before the introduction of the spiders (figure 3, before spiders; one-sample Wilcoxon signed-rank test, *Z*=−2.919, *p*=0.004). The mean number of highly rewarding flowers chosen in the last 30 choices was significantly greater than in the first 30 choices (figure 3, before spiders; Wilcoxon signed-rank test, *Z*=−2.53, *p*=0.011). The bees were thus able to learn to use the odour cues to choose the highly rewarding flowers in the absence of spiders. The bees took an average of 108.9 (±13.3 s.d.) choices from 3.3 (± 0.8 s.d.) bouts to learn the task.

After the spiders were introduced, bees still chose highly rewarding flowers significantly above chance in both the first 30 choices after spiders (figure 3, after spiders; one-sample Wilcoxon signed-rank test, *Z*=−2.821, *p*=0.005) and the last 30 choices (figure 3, after spiders; one-sample Wilcoxon signed-rank test, *Z*=−2.81, *p*=0.005). The number of ‘dangerous’ flowers chosen was also significantly below chance levels in both the first 30 choices (figure 3, after spiders; one-sample Wilcoxon signed-rank test, *Z*=−2.509, *p*=0.012) and the last 30 choices (figure 3, after spiders; one-sample Wilcoxon signed-rank test, *Z*=−2.641, *p*=0.008) made after the introduction of the spiders. The bees were thus able to perform both the olfactory task (differentiation of the highly rewarding flowers) and the visual task (avoiding the spiders) simultaneously.

### 3.4 Comparisons across experiments

Comparing the mean number of highly rewarding flowers chosen before the introduction of the spiders, we found a significant main effect of the point in the time course (first 30 or last 30 choices) (Friedman’s two-way ANOVA, *χ*^2^=17.9,*p*<0.001) but no significant main effect of modality condition (intramodal experiment or crossmodal experiment) (Friedman’s two-way ANOVA, *χ*^2^=0,*p*=1).

After the introduction of the spiders, we found that there was a significant main effect of the modality condition on the mean number of highly rewarding flowers chosen (Friedman’s two-way ANOVA, *χ*^2^=5.67, *p*=0.017) and no main effect of the point in the time course (Friedman’s two-way ANOVA, *χ*^2^=0, *p*=0.96).

These results show that before the introduction of the spiders, bees learnt the tasks equally well in both the intramodal and crossmodal conditions. However, after the introduction of the spiders, the performance of the bees differed in the intramodal and crossmodal conditions.

## 4. Discussion

We tested bees’ ability to perform two tasks simultaneously in two different experiments: one intramodal and one crossmodal. We found that when the two tasks faced by the bees were in the same modality, bees failed to perform both simultaneously. When, however, one task was a visual task and one olfactory, bees managed to perform both tasks simultaneously. These results argue for attentional or cognitive resources in the bee brain being allocated separately specific to each modality. Furthermore, the difficulty of the visual and odour tasks—as measured by the number of choices taken to learn each task and the level of accuracy reached after learning—did not differ, arguing against the possibility that the odour task was merely easier to perform than the visual task. This further indicates that the difference between the crossmodal and intramodal experiments reflects processing limitations in the latter that are absent in the former. The similarity in task performance also rules out the possibility of overshadowing where, for example, a more salient odour stimulus might have prevented learning of a less salient visual stimulus. It is also interesting that the task difficulty is similar for both tasks given that the two odours used were very dissimilar. The difficulty of the odour task could be due to a combination of factors contributing to lower levels of odour molecules available to the bees. These include using low concentrations and volumes of the scents in larger vials, regular ventilation and lack of direct access to the scent cues.

One interesting question that arises from our results is what evolutionary advantage is conferred by having separate resources for processing visual and odour tasks. It appears that this could be one way of facilitating the simultaneous performance of different tasks, as borne out by our experiments. Thus, bees should be better able to avoid predators while foraging as they can still choose high-rewarding flowers based on odour cues while spotting spiders visually. We should also expect them to be able to do the reverse—detecting predators through smell while using visual cues to spot high-reward flowers. Thus, this ability to process modalities separately seems to be suited to the ecology of the bees and could have been selected for to give them an advantage while performing more than one task.

Recent studies have begun revealing the mechanisms of visual attention in insects [38,43]. Spatial cues have been shown to direct orientation in flies, dragonflies and hoverflies [44–46] and serve as priming cues that bias attention in flies [47]. The learnt saliency of an object can also bias both the behaviour and neural activity of flies [48]. Other studies have also suggested that different insects have specific visual search mechanisms, with honeybees perhaps being restricted to ‘serial’ visual search while bumblebees manage to have ‘parallel’ visual search similar to that seen in humans [49,50]. Few studies have, however, investigated insect attention to multiple target stimuli [14] or multimodal stimuli [33,34]. One study that has investigated attention to multiple targets in bees found that bees could attend to multiple targets with divided attention if the distractor targets were associated with aversive taste like quinine [13], albeit at the expense of substantially increased inspection times. Our study extends these results to show that bees can also deploy attention across multiple targets if they are present in multiple modalities.

The candidate structures in the insect brain that have been implicated in attention-like processes are the mushroom bodies [51,52]. Flies with defects in these fail to show the abrupt changes in saliency-based choices between two stimuli seen in non-defective flies and instead show a more graded response [51]. Furthermore, they appear to be more easily distracted from visual tasks by olfactory stimuli [52]. This argues for the mushroom bodies probably mediating the kinds of learnt responses we see where the processing of olfactory cues does not interfere with that of visual cues. It is also interesting to note that the visual and olfactory neural pathways in bees stay largely separate peripherally and input from both these pathways is received by specialized regions of the mushroom bodies [53], where the lip region receives olfactory input from the antennal lobes, the projections from the optic lobes terminate in the collar, and only the basal ring of the mushroom bodies receives both visual and olfactory inputs [43,54–56]. Our crossmodal tasks could thus be processed in these separate specialized regions without interfering with each other, while the visual tasks could both involve processing in similar regions resulting in intramodal interference.

Our results are in agreement with studies in humans that showed that for certain tasks, it was possible to attend to information in different modalities without any detriment to processing. Treisman & Davies [18] showed that there was greater interference in the detection of a target when stimuli were present in the same modality (visual or auditory) compared with when they were present in different modalities. Their results were supported by other studies that showed a similar absence of crossmodal interference in the perception of stimuli despite there being interference within a given modality [20,23,28]. Some researchers have argued that these results suggest separate attentional resources for different modalities [23,24,57]. This is not, however, true for all tasks and other studies have shown that for several other tasks there is still a decrease in performance when the two tasks are present in different modalities [3,25,26,29]. One explanation for the differences between these results might be that different tasks divide attention differently across and within modalities. Dividing attention across modalities might, for example, be costly when one has to recognize a target but not when one has to detect a target [21]. In addition, some of the crossmodal decrements in processing could be related more to decision making rather than attention *per se* [22]. Lavie [58] and Lavie & Tsal [59] also make the interesting point that attentional load could influence the degree of interference with processing. They make a case for irrelevant information (e.g. information from another modality) interfering in attention only when the entire processing pool allocated for relevant stimuli is not exhausted. Thus, perhaps tasks within one modality that are harder rather than easier to perform are less likely to show a decrease in performance due to crossmodal interference. Taken together, these results argue that crossmodal influences on attentional resources could depend on the specific tasks studied. Therefore, while our results clearly show the possibility of modality-specific attention in bumblebees, it would be important for future studies to investigate crossmodal attention with tasks that differ both in type and difficulty to bring to light when attention-like sensory processing in bees is modality-specific and when it is not.

## Supplementary Material

Choices of highly or low rewarding flowers made by the bees in both the intramodal and crossmodal conditions. 0.3 and 0.5 refer to choices of a low or highly rewarding flower respectively. S refers to a choice of highly rewarding flower with a spider on it.

## Data accessibility

The datasets supporting this article have been uploaded as part of the electronic supplementary material.
